# Machine learning algorithm to characterize antimicrobial resistance associated with the International Space Station surface microbiome

**DOI:** 10.1186/s40168-022-01332-w

**Published:** 2022-08-24

**Authors:** Pedro Madrigal, Nitin K. Singh, Jason M. Wood, Elena Gaudioso, Félix Hernández-del-Olmo, Christopher E. Mason, Kasthuri Venkateswaran, Afshin Beheshti

**Affiliations:** 1 Jeffrey Cheah Biomedical Centre, Wellcome-MRC Cambridge Stem Cell Institute, University of Cambridge, Cambridge Biomedical Campus, Puddicombe Way, Cambridge, CB2 0AW UK; 2 Present Address: European Molecular Biology Laboratory, European Bioinformatics Institute, EMBL-EBI, Hinxton, CB10 1SD UK; 3 Biotechnology and Planetary Protection Group, Jet Propulsion Laboratory, California Institute of Technology, Pasadena, CA 91109 USA; 4 Department of Artificial Intelligence, Computer Science School, Universidad Nacional de Educación a Distancia (UNED), 28040 Madrid, Spain; 5 Department of Physiology and Biophysics, Weill Cornell Medicine, New York, NY 10065 USA; 6 The HRH Prince Alwaleed Bin Talal Bin Abdulaziz Alsaud Institute for Computational Biomedicine, Weill Cornell Medicine, New York, NY 10065 USA; 7 The WorldQuant Initiative for Quantitative Prediction, Weill Cornell Medicine, New York, NY 10065 USA; 8 The Feil Family Brain and Mind Research Institute, Weill Cornell Medicine, New York, NY 10065 USA; 9 KBR, Space Biosciences Division, NASA Ames Research Center, Moffett Field, CA 94035 USA; 10 Stanley Center for Psychiatric Research, Broad Institute of MIT and Harvard, Cambridge, MA 02142 USA

**Keywords:** ISS, Metagenomics, Antibiotic resistance, Machine learning, Space Omics, Microbiome, Built-environment, Microbial Tracking-1, NGS

## Abstract

**Background:**

Antimicrobial resistance (AMR) has a detrimental impact on human health on Earth and it is equally concerning in other environments such as space habitat due to microgravity, radiation and confinement, especially for long-distance space travel. The International Space Station (ISS) is ideal for investigating microbial diversity and virulence associated with spaceflight. The shotgun metagenomics data of the ISS generated during the Microbial Tracking–1 (MT-1) project and resulting metagenome-assembled genomes (MAGs) across three flights in eight different locations during 12 months were used in this study. The objective of this study was to identify the AMR genes associated with whole genomes of 226 cultivable strains, 21 shotgun metagenome sequences, and 24 MAGs retrieved from the ISS environmental samples that were treated with propidium monoazide (PMA; viable microbes).

**Results:**

We have analyzed the data using a deep learning model, allowing us to go beyond traditional cut-offs based only on high DNA sequence similarity and extending the catalog of AMR genes. Our results in PMA treated samples revealed AMR dominance in the last flight for *Kalamiella piersonii*, a bacteria related to urinary tract infection in humans. The analysis of 226 pure strains isolated from the MT-1 project revealed hundreds of antibiotic resistance genes from many isolates, including two top-ranking species that corresponded to strains of *Enterobacter bugandensis* and *Bacillus cereus*. Computational predictions were experimentally validated by antibiotic resistance profiles in these two species, showing a high degree of concordance. Specifically, disc assay data confirmed the high resistance of these two pathogens to various beta-lactam antibiotics.

**Conclusion:**

Overall, our computational predictions and validation analyses demonstrate the advantages of machine learning to uncover concealed AMR determinants in metagenomics datasets, expanding the understanding of the ISS environmental microbiomes and their pathogenic potential in humans.

Video Abstract

**Supplementary Information:**

The online version contains supplementary material available at 10.1186/s40168-022-01332-w.

## Background

According to the World Health Organization, the widespread use of antibiotics worldwide and the slow discovery of major types on antibiotics in the last thirty years has made antibiotic resistance one of the biggest threats to human health, food security, and development [58]. Accordingly, with NASA setting the course to return to the Moon with the Artemis mission and eventually venture out to Mars, maintaining the health of astronauts during long-term spaceflight is of paramount importance [1]. One area of particular concern is the reported increase in virulence and antibiotic resistance of microorganisms in space experiments [4, 30, 36, 49, 51, 56, 61]. Combined with a depressed or altered immune response in astronauts [25, 46], there is an increased risk of opportunistic microbial infection. Spaceflight promotes biofilm formation [32], and bacteria cultured from astronauts during flight were more resistant than isolates obtained from the same individual either pre- or post-flight [50]. Mutations also occurred more frequently in long-term spaceflights [24]. An alternative non-mutually exclusive hypothesis to increased virulence or microbial resistance to antibiotics is that spaceflight conditions might alter the stability of pharmaceuticals [23]. In any case, bacterial infections might be more challenging to treat in space.

The International Space Station (ISS) is a closed-built environment with its own environmental microbiome shaped by microgravity, radiation, and limited human presence [53]. We and others have shown that microbiomes are dynamic, diverse and sometimes intertwined at the ISS. Be et al. [8] analyzed antibiotic resistance and virulence genes from dust and vacuum filter samples of ISS (treated with propidium monoazide, or PMA), demonstrating that human skin-associated microbes impact the ISS microbiome. Indeed, the skin and intestinal microbiomes of astronauts that spent 6 to 12 months in the ISS have been shown to be altered [55]. In addition, the salivary microbiome of astronauts changed as a result of spaceflight, potentially activating microbes that promote viral replication [52] and altering the abundance of some antimicrobial resistance (AMR) genes [35]. The ISS itself also presents specific core microbiome signatures on its surfaces that we characterized recently using shotgun metagenome and amplicon sequencing [14, 43, 51], analogous to microbiome signatures found in specific geographies on Earth [18].

Further analyses across several missions have revealed that the microbiome of the crew's skin resembled those of the surfaces inside the ISS collected by the crewmember on the same flight [5]. To better understand the composition of these bacterial populations we and others have characterized shotgun whole-genome sequencing (WGS) of several ISS microorganisms [10, 11, 45]. Although most of them have been found to be non-pathogenic to humans, there are exceptions such as antibiotic-resistant *Enterobacter bugandensis* strains that could have an increased chance of pathogenicity [44].

**Fig. 1:**
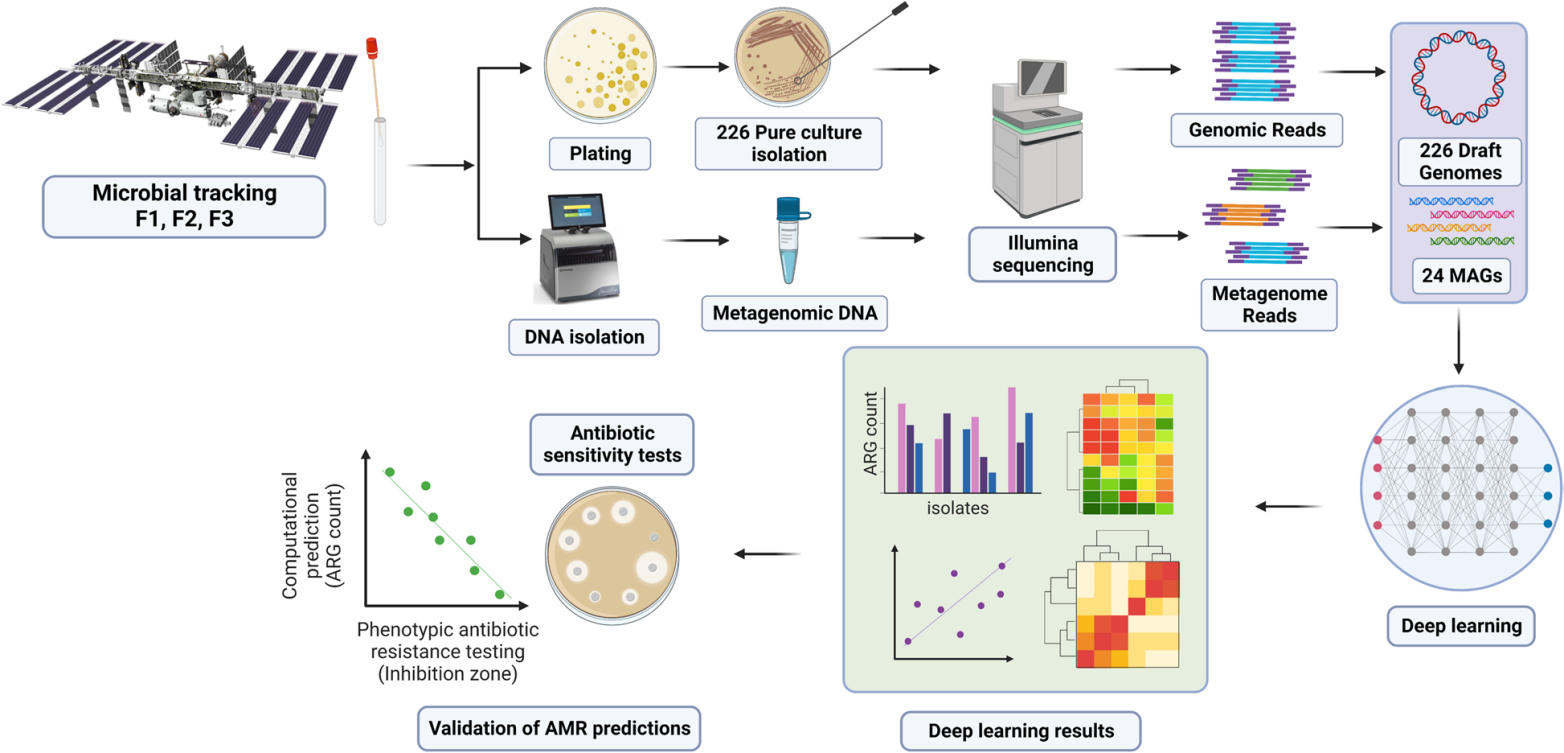
Overview of sample collection and data analysis for the characterization of antibiotic resistance at the ISS using deep learning. The data are processed in a step-wise fashion including data QC, mapping, quantification, and matching to time of collection and mission. The figure has been generated using BioRender (http://biorender.com)

Computational analyses of microbiome data collected in Earth have shown that AMR can be predicted from genomic sequence of pure cultures alone [29, 47], but a consensus approach on the best way to detect AMRs in metagenomic datasets has yet to be established [41]. Generally, predictions are restricted to high identity (high sequence similarity to databases) cut-offs, requiring a ‘best-hit’ on an appropriate AMR database with a sequence identity greater than 80% by many programs such as ResFinder [60]. Although the ‘best-hit’ approach has a low false-positive rate, the false-negative rate can be very high, and a large number of actual Antibiotic Resistance Genes (ARGs) are predicted as non-ARGs, thus concealing the identification of potentially functional ARGs [3]. Another method of identification is to link the immune repertoire of the astronaut to the peptides of the microbes on the ISS, but this requires complex coordination with crew sampling and is rare [19]. However, it has been shown recently that deep learning, a class of machine learning algorithms, can expand the catalog of AMR genes and increase the accuracy of the predictions based on metagenomic data [3, 13, 27]. We then hypothesized that the characterization of AMR from sequencing data at the ISS could be investigated from an artificial intelligence perspective using a robust deep learning framework. For that, we analyzed whole-genome sequences of 226 pure strains (cultivable microbes), metagenome sequences of 21 environmental samples, and 24 MAGs retrieved from PMA treated samples (Fig. 1) using the supervised deep learning approach proposed by Arango-Argoty et al. [3], which has shown high sensitivity for detection of AMR genes in an independent benchmark [57].

## Results

### Predictions based on short metagenomics sequences and ORFs partly overlap with previous analyses and reveal new AMR determinants at the ISS surface microbiome

**Fig. 2:**
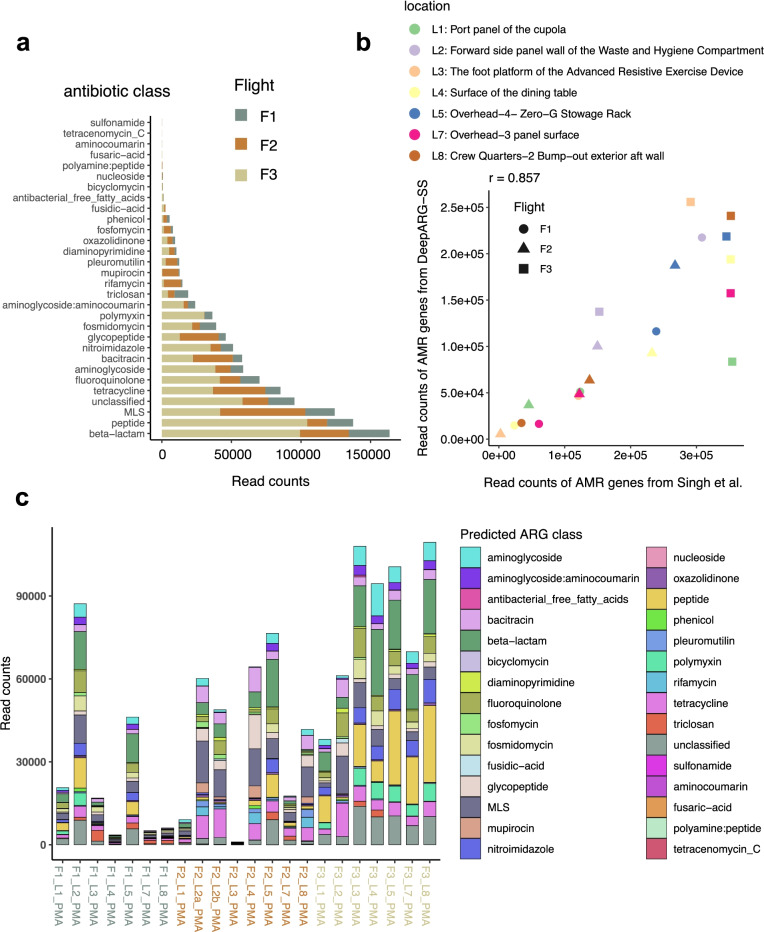
Prediction of ARGs using a pre-trained DeepARG-SS model. **a** Distribution of ARG read counts across antibiotic classes for the three flights (F1, F2, F3). **b** Correlation of read counts found by DeepARG-SS and those in Singh et al. [43]. Pearson's product-moment correlation *r* = 0.86, (*p* = 6.879e−07) for the three flights and their locations. **c** Read counts of ARG class across flights for each location for PMA-treated samples in Singh et al. [43, 44]. The antibiotic class (*multi-drug*) is not shown. Results are for ARGs with probability > 0.8

The first shotgun metagenome sequencing of intact microbial cells (Propidium monoazide-PMA treated) without whole-genome amplification was performed by Singh et al. [43]. There, samples were taken in 8 locations across three flights (F1, F2, F3) during a period of 12 months. A detailed description of sampling procedures and locations, species diversity and functional characterization can be found in Singh et al. [43]. To deploy a deep learning approach for predicting antibiotic resistance genes from metagenomic data, we used DeepARG, a computational resource proven to be more accurate than traditional approaches [3]. We first run DeepARG-SS (DeepARG for short reads) using the recommended prediction probability cut-off of 0.8 to obtain read counts of AMR genes (Fig. 2a). As in the seminal paper [43], quantification of antibiotics associated with AMR revealed ‘beta lactams’ ranking first and ‘peptide’ second, and generally more AMR reads counts observed in Flight 3 (F3) than in previous two flights (Fig. 2a). However, reads counts in certain antibiotics such as pleuromutilin, mupirocin and rifamycin were found largely in Flight 2 (Fig. 2a). Our read counts correlate (*r* = 0.86, *p* = 6.879e−7; Pearson’s product-moment correlation) with read counts obtained for antimicrobial resistance by Singh et al. [43] (Fig. 2b). Taken together, these suggest a partial overlap with results obtained in Singh et al. [43] analyzed using the traditional approach.

While more AMR reads counts were found in Flight 3, we also observed variability between the different locations and flights, and an increasing number of read counts associated with time. For instance, location 4 (L4, surface of the dining table) increased the number of AMR reads counts with successive flights (Fig. 2b, c). While resistance to ‘beta lactams’ was evenly distributed across flights and locations, resistance to ‘polymyxin’ and especially ‘peptide’ represents a more significant proportion of AMR counts in locations of Flight 3 (Fig. 2c). In addition, we also observed the widespread presence of reads related to Macrolides, Lincosamides, Streptogamines (MLS), and tetracycline resistance.

**Fig. 3:**
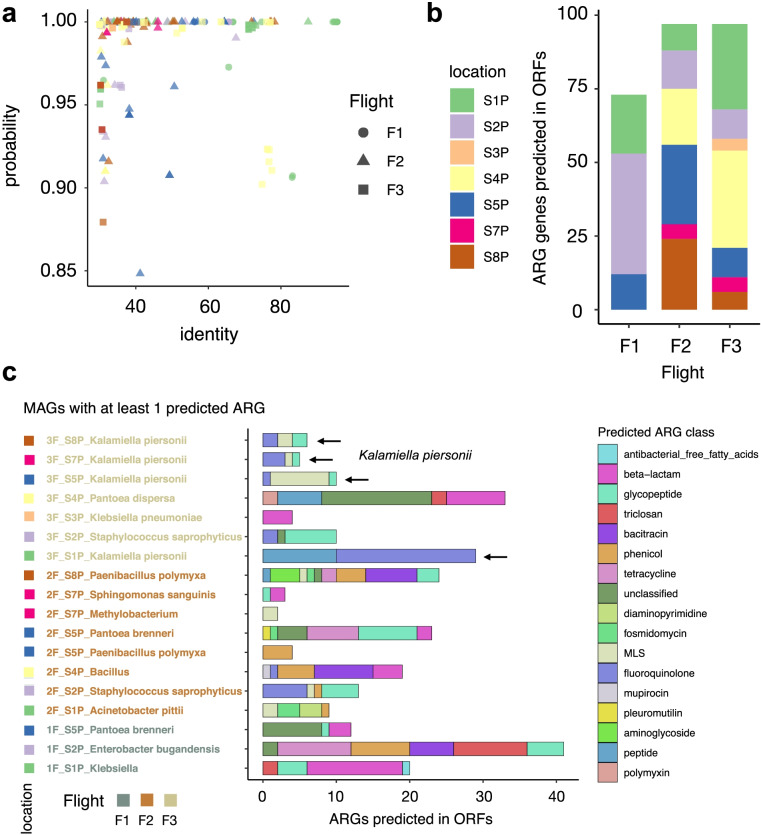
ARGs detected in ORFs in metagenome-assembled genomes (MAGs) from PMA-treated samples. **a** Distribution of DeepARG classification probability and best-hit identity in MAGs retrieved from the ISS. **b** Total number of ARGs predicted for each flight and location. **c** Number of ARGs precited for each MAG. Most common antibiotic class (*multi-drug*) not shown. The black arrows indicate *Kalamiella piersonii*

To investigate the possible association between AMR patterns and specific microbes, we assembled the short reads into Metagenome-Assembled Genomes (MAGs; see Methods), identified their Open Reading Frames (ORFs), and repeated the prediction of ARGs using DeepARG-LS [3]. Figure 3a shows the distribution of DeepARG classification probabilities and best-hit identity of ARGs in MAGs from the ISS. As we can retrieve highly probable ARGs (probability > 0.8) presenting low sequence identity (for many ARGs, identity is < 40%), this method is likely more advantageous than using the ‘best-hit’ approach only. Compared to DeepARG-SS results obtained previously, the analysis of MAGs did not reveal significant differences in the number of ARGs predicted in the ORFs for the different flights (Fig. 3b). However, interestingly the results show a smaller number bacterial species having ARGs in Flight #1 (F1) when compared to Flights #2 and #3 (Fig. 3b, c) (data is shown for MAGs with at least 1 predicted ARG; the total number of MAGs analyzed is 24). Specifically, the number of locations is smaller in Flight 1 (*n* = 3) than in F2 (*n* = 6) and F3 (*n* = 7) (Fig. 3b)**.** Many ARGs were identified in *Kalamiella piersonii* MAGs in multiple locations during F3, showing AMR patterns related to (glyco)peptide, fluoroquinolone and MLS (Fig. 3c). Of note, the *K. piersonii* strain closely related to one found at the ISS has been associated to human urinary tract infection [40]. The potentially very pathogenic microbe *E. bugandensis* was found in location 2 (forward side panel wall of the Waste and Hygiene Compartment) in Flight 1, presenting more than 40 ARGs. In addition, in the original study, *Pantoea* species were found to be the dominant genus in samples in 5 out of 7 locations sampled from Flight 3, especially at location 5 (surface rack). In our re-analysis, we observed *Pantoea brenneri* and *Pantoea dispersa* having ARGs related to beta-lactams and peptide [43], as well as to triclosan and polymyxin resistance.

Overall, our results partially agree with earlier findings while providing new insights into previously unobserved antibiotic resistance classes (of the 30 antibiotic resistance categories included in the model). Specifically, the re-analysis of short sequences and MAGs from the ISS reveals dominance of *K. piersonii* antibiotic resistance in different locations of Flight 3 (Fig. 3c).

### Distribution of antibiotic resistance genes in scaffolds of Microbial Tracking-1 strains isolated from the ISS

**Fig. 4:**
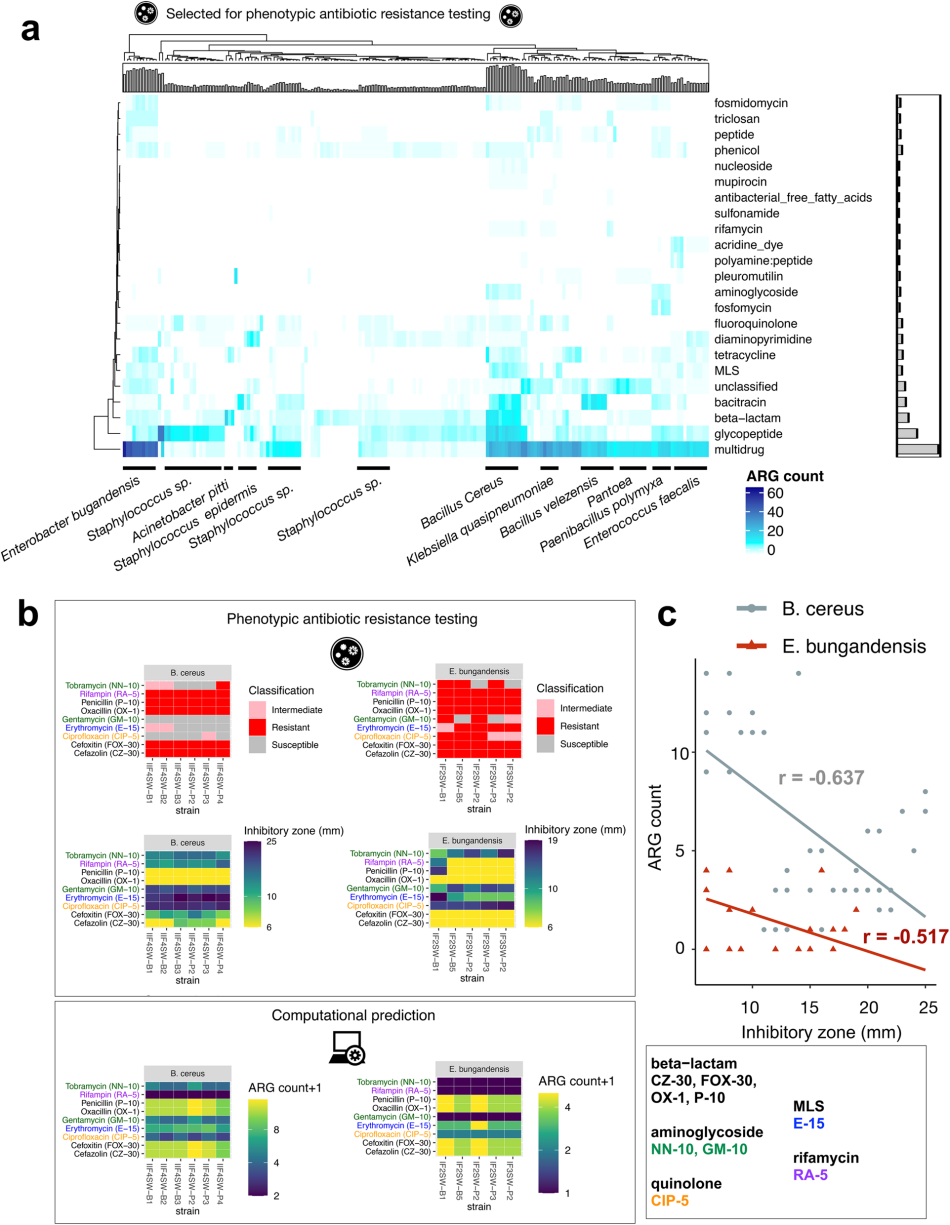
Heatmap and clustering of ARG counts detected in MT-1 pure strains isolated from the ISS and AST validations. **a** Heatmap with ARG count. The barplots illustrate the number of ARGs across rows and across columns. Species were identified using BLAST. Only ARGs with probability > 0.8 were considered, as recommended. **b** Antibacterial susceptibility tests (AST) on *E. bugandensis* and *B cereus* strains for several antibiotics (top), and comparison with machine learning predictions shown in (**a**) (bottom). **c** Scatterplot of zone of inhibition value (in mm.) and ARG count shown in (**b**), together with a linear model fit. Pearson's product-moment correlation values are indicated

We then applied DeepARG-LS to 226 Microbial Tracking-1 (MT-1) isolates (Mason and Venkateswaran labs, published and unpublished WGS of MT-1 pure strains isolated from ISS environment). We found a range of 2 to 92 ARGs in 184 out of 226 isolates (Fig. 4a; Table S1). This machine learning approach allowed us to go beyond the traditional cut-off based only on high sequence DNA similarity (Figure S1). These results suggest a widespread presence of potential ARGs in the isolates, with ‘multi-drug’ class being first, followed by glycopeptides, beta-lactams, bacitracin, and tetracyclines. The ‘multi-drug’ antibiotic class was defined by aggregating several antibiotic names from the CARD and ARDB databases (efflux, multi-drug and na_antimicrobials). We then used BLAST to match isolates showing AMR sequences predicted by DeepARG to microbial species (Fig. 4a) and identified *Bacillus cereus* and *E. bugandensis*, which were previously profiled organisms on the ISS [44, 54] as the top 2 ranking species with a high number of ARGs. We have previously shown that five *E. bugandensis* isolates were almost equivalent to nosocomial earth isolates showing resistance to multi-drug antibiotic compounds, fluoroquinolones, and fosfomycin [44]. In addition, *E. bugandensis* strains were shown to be resistant to 9 antibiotics [51]. Our results reinforce the potential pathogenicity of this microbe. Nonetheless, antimicrobial resistance was not examined for *B. cereus* strains in Venkateswaran et al. [54]. *B. cereus* is a food poisoning microorganism that might be a concern for crew members' health. In addition, we found novel ARGs associated with other species such as *K. pneumoniae*, *Pantoea*, *Paenibacillus polymyxa*, *Bacillus velezensis*, *Enterococcus faecalis*, *Sphingomonas,* and, with a lower number of ARGs, several species of *Staphylococcus*. *E. faecalis* virulence was previously shown to be affected by microgravity [28]. We then used the tool Prokka [42] to fully annotate the bacterial isolates, finding as expected that the number of coding sequences, but not the number of ARGs, increased in proportion to genome sizes (Figure S2a). Then, we ran the pan-genome analysis tool Roary [38] to compare isolates of *E. bugandensis* (10) and *B. cereus* (10) finding that the core set of genes was highly conserved among strains of the same species (Figure S2b).

To experimentally validate machine learning predictions on previously unobserved AMR patterns above, we performed Antibacterial Susceptibility Tests (AST) for the species found to be potentially most pathogenic, in our case *E. bugandensis* and *B. cereus* as they have a higher number of ARGs (Table S1; Fig. 4a). For that, we use disc diffusion on strains isolated at the ISS for the following antimicrobials: Cefazolin (beta−lactam), Cefoxitin (beta−lactam), Ciprofloxacin (quinolone), Erythromycin (MLS), Gentamycin (aminoglycoside), Oxacillin (beta−lactam), Penicillin (beta−lactam), Rifampin (rifamycin), and Tobramycin (aminoglycoside) (Fig. 4b). The prediction patterns closely matched the AST results (Fig. 4b), although DeepARG failed to detect Rifampin resistance, especially for *E. bugandensis*.

Although different antibiotics have different inhibitory zone cut-offs for a strain to be considered as resistant (Table S2), remarkably we found an inverse correlation between the zone of inhibition and ARG count for *B. cereus* (*r* = − 0.637, Pearson's product-moment correlation, *p* = 2.2e−7) and *E. bugandensis* (*r* = − 0.517; *p* = 0.0002765) (Fig. 4c), demonstrating the applicability and high accuracy of computational prediction of AMR for microbiome data obtained in space.

## Discussion

Many ARGs that present high probability but low sequence identity to known sequences will be missed using traditional ‘best-hit’ approaches that require a high degree of sequence identity. To solve this, computational methods have been developed to identify AMR in genomes and metagenomes [3, 9, 16, 33, 41]. Despite these developments, a consensus approach to detect AMR in metagenomics datasets is yet to be defined [41]. The objective of this study was to identify the AMR genes associated with cultivated strains and metagenomes generated from the ISS environmental surfaces using an accurate deep learning approach (Fig. 1).

Firstly, we re-analyzed shotgun metagenome sequences of 21 environmental samples that were treated with PMA (viable microbes), and their associated 24 MAGs retrieved from the PMA-treated samples. The re-analysis showed increased read counts associated with AMR and in more locations for flight 3 when considering MAGs (Fig. 2). This could be explained due to the ISS crew being replaced during Flight 3. The abundance of *Enterobacteriaceae* in Flight 3 was discussed in Singh et al. [43]. We have not observed any differences between early vs. late ISS microbiome cultures. For example, *Enterobacter bugandansis* strains were isolated from F2 and F3 sample sets, but the genome comparison and phenotype analyses revealed limited change, with a maximum of 15 SNPs among ISS isolates [44]. In addition, *K. piersonii* spread across four different locations (L1, L5, L7, L8) at Flight 3, presenting resistance to specific antibiotics (glyco/peptide, fluoroquinolone and MLS) (Fig. 3c). We have previously isolated strains from Locations 1, 2, 5, 6, and 7, defining a novel bacterial genus from the ISS samples [45]. While *K. piersonii* do have virulence genes in the genome, a dichotomy was found as disc diffusion tests revealed multi-drug resistance, while the PathogenFinder algorithm predicted *K. piersonii* strains as non-human pathogens. All seven *K. piersonii* isolates were resistant to cefoxitin (beta_lactam class in DeepARG), erythromycin (MLS), oxacillin (beta_lactam), penicillin (beta_lactam), and rifampin. At the same time, all strains were susceptible to cefazolin, ciprofloxacin (quinolone), and tobramycin (aminoglycoside) [45]. The DeepARG database does not include some of these antibiotics, but we found AMR sequences related to resistance to (glyco)peptide, fluoroquinolone, and MLS, validating some previous results. Therefore, PathogenFinder [17] results in Singh et al. [45] suggesting *K. piersonii* as a non-human pathogen should be treated with caution. Furthermore, the strain YU22 (closest match is IIIF1SW-P2^T^ detected as ISS) isolated in urine microbiome of a kidney stone patient has shown to be an uropathogenic bacteria, showing many virulence factors that are needed for host cell invasion and colonization [40].

Secondly, the whole-genome sequences (WGS) of 226 pure strains (cultivable microbes) were analyzed to identify AMR genes (Fig. 4a). We found the human pathogens *E. bugandensis* and *B. cereus* presenting many potential ARGs in the MT-1 scaffolds. Up to five strains isolated from the ISS have been closely related to the type strain EB-247^T^ and two clinical isolates (153_ECLO and MBRL 1077) and share similar AMR patterns [44]. One hundred twelve genes were found to be involved in virulence, disease, and defence in the ISS strains [44]. Our re-analysis confirms the *multi-drug* resistance (MDR) to antibiotics for the ISS isolates, which is the highest among all the isolates. Our previous research uncovered the presence of genes associated with MDR efflux pump [44], belonging to RND (resistance, nodulation and cell division) protein family, which are reported to be the major contributors of resistance to antibiotic and other toxic compounds to the bacteria [20]. MDR has been reported to play role in the physiological function and confer resistance to substances like host defense molecule and bile, which can lead to pathogenicity in humans [48]. Unlike in Singh et al. [43], we found fluoroquinolone resistance low, and null for fosfomycin. Conversely, *B. cereus* is a gram-positive bacterium commonly found in food. After infection, most emetic patients recover within 24 hours, but in some cases, the toxin can be fatal via a fulminant hepatic failure [22, 34]. Overall, *multi-drug* resistance was found widespread in many microbes.

Third, phenotypic antibiotic resistance testing data obtained from traditional antibiotic tests generated for biosafety level 2 strains were compared with the computational approaches that predicted the presence of the AMR genes, showing an excellent agreement for the antibiotics tested (Fig. 4b, c). A disadvantage of the deep learning model developed by Arango-Argoty et al. [3] is that the prediction can disentangle the family of antibiotics but not specific compounds.

Many studies have shown the association between several microorganisms (bacterial, as well as phage and non-phase viral sequences) and several cancer features. Although it is unclear whether this corresponds to correlation or causation, the microbiome can undoubtedly be used as a cancer biomarker. For instance, certain strains of *Fusobacterium *sp. can be utilized as an independent diagnostic assay for colon cancer [62]. Therefore, a better understanding of the microbial communities and their degree of pathogenicity in surface-human microbiomes in space could also be useful for human health monitorization with detection and prognostic values in long term space travel. Rather than the gather-and-return sampling model currently used for the ISS, new developments in sequencing technologies in combination with Artificial Intelligence will allow for efficient analysis onboard the ISS and in long-duration space missions.

We are currently collecting more data for Microbial Tracking-2 (MT-2) and MT-3 missions. We plan to extend the AMR catalog, characterize microbial diversity, and monitor the evolution of AMR in longer time periods to discover new factors involved in pathogenicity of microorganisms exposed to space conditions.

## Methods

### Metagenome-Assembled Genomes (MAGs) methodology

The paired-end 100-bp metagenomic reads from NCBI Short Read Archive (SRA) under the bio-project number PRJNA438545 were processed with Trimmomatic [12] to trim adapter sequences and low-quality ends, with a minimum Phred score of 20 across the entire length of the read used as a quality cut-off. Reads shorter than 80 bp were removed after trimming. Remaining high-quality reads were subsequently assembled using metaSPAdes [37]. Contigs were binned using Metabat2 v2.11.3 [31]. Recovered genomes were evaluated with CheckM [39], and a recovered genome was considered good with at least 90% completeness and at most 10% contamination. Each genome was subsequently annotated with the help of Rapid Annotations using Subsystems Technology (RAST), and near identifications were predicted [6].

### Sample collection for Microbial Tracking 1 mission

During the microbial tracking investigation to characterize airborne and surface-associated microbial population aboard the International Space Station, samples were collected from ISS locations, and ground samples were collected from the Crew resupply vehicle. A sterile polyester wipe premoistened with Phosphate buffered saline (PBS) was used to collect the samples from various areas across these ISS locations, and the details and description of the sample collection and cultivation have already been reported in Checinska Sielaff et al. [14] and Singh et al. [43]. Pure isolates were selected and sub-cultured, and the sub-cultures were sequenced.

### Isolates from Microbial Tracking 1 mission

To create the whole-genome sequences (WGS) of these strains, shotgun libraries were prepared using the Illumina Nextera Flex protocol [44], using NovaSeq 6000 S4 flow cell 2150 paired-end (PE) sequencing. Verification of the quality of the raw sequencing data was carried out using FastQC v0.11.7 [2]. Quality control for adapter trimming and quality filtering were performed using fastp v0.20.0 [15], and then SPAdes v3.11.1 [7] was used to assemble all the cleaned sequences. Fastp quality control was based on the following three parameters: (i) correction of mismatches in overlapped regions of paired-end reads, (ii) trimming of autodetected adapter sequences, and (iii) quality trimming at the 59 and 39 ends. To determine the quality of the assembled sequences, the number of contigs, the N50 value, and the total length were calculated using QUAST v5.0.2 [26]. Default parameters were used for all software. The average nucleotide identity (ANI) [59] was calculated using OrthoANIu by comparing each of the scaffolds to the WGS of the respective type strains.

### Identification of ORFs in microbial DNA sequences

Glimmer (Gene Locator and Interpolated Markov ModelER) v3.02 was used with default parameters to identify the coding regions and distinguish them from non-coding DNA in MAGs and MT-1 scaffolds that could be used as an input in DeepARG-LS. Minimum gene length was indicated as 50 bp (‘glimmer3 -g50’). Glimmer reads DNA sequences in a FASTA file format and predicts genes in them using an Interpolated Context Model [21].

### Prediction of antibiotic resistance genes in short reads and full-gene length sequences

DeepARG version 2 [3], a deep learning-based approach for predicting ARGs and annotation, was run with the ‘--reads’ option (DeepARG-SS) for NGS reads and the ‘--genes’ option (DeepRG-LS) for longer gene-like sequences obtained with Glimmer. The DeepARG model consists of four dense hidden layers of 2000, 1000, 500, and 100 units that propagate a bit score distribution. The output layer of the deep neural network consists of 30 units that correspond to the antibiotic resistance categories (102 antibiotics consolidated into 30 antibiotic categories). The model was trained with a curated database of 14,933 genes from three databases (CARD, ARDB, and UNIPROT) [3]. Default options were used: 50% minimum percentage of identity to consider, significance of the prediction probability cut-off of 0.8 as recommended [3], and E-value of alignments (default 1e−10). The software was downloaded from https://bitbucket.org/gusphdproj/deeparg-ss.

### Microbial nucleotide BLAST

Nucleotide-Nucleotide BLAST 2.10.1+ (https://blast.ncbi.nlm.nih.gov/Blast.cgi) was used to identify microbial species associated to MT-1 scaffolds. Sequences producing significant alignments were ranked and the species associated to maximum Score (bits) and minimum *E* value was deemed as the closest match.

### Gene annotations and pan-genome analysis of MT-1 scaffolds

A Docker image with the software tool Prokka v1.14.5 [42] was pulled and used with default parameters to annotate bacterial isolates. The master annotations in GFF3 format for the 226 isolates have been deposited at the Zenodo platform [DOI: 10.5281/zenodo.6518836]. Using the annotated assemblies in GFF3 format for *E. bugandensis* and *B. cereus* strains, Roary v3.13.0 was run with default parameters to compare isolates based on genes and the number of isolates they are present in.

### Phenotypic antibiotic resistance testing

Disc assays experiments were performed and reported in Urbaniak et al. [51]. The isolates were streaked from glycerol stocks onto R2A plates. A single colony was inoculated into 5 mL Tryptic Soy Broth (TSB) and grown overnight at 30°C. Aliquots of 100 μL were plated on TSA. Agar diffusion discs (BD BBLTM Sensi-DiscTM, Franklin Lakes, NJ) were placed aseptically on a plate and the strains were incubated at 37 °C for 24 h. The tested antibiotics included: 30-μg cefazolin (CZ-30); 30-μg cefoxitin (FOX-30), 5 μg ciprofloxacin (CIP-5), 15 μg erythromycin (E-15), 10-μg gentamicin (GM-10), 1-μg oxacillin (OX), 10-μg penicillin (P-10), 5-μg rifampin (RA-5), and 10 μg tobramycin (NN-10). The diameter of inhibition zones was measured for each antibiotic disk and recorded in millimeters. The resistance results were compared with the zone diameter interpretive charts provided by the manufacturer. When the spontaneous mutants were present in response to some antibiotics, they were isolated, subcultured and tested for the specific antibiotic resistance.

### Data availability

Raw metagenomics reads from three flights on multiple locations were downloaded from NASA GeneLab repository https://genelab.nasa.gov (GLDS-69). The ISS MAGs datasets are available at the SRA database under the accession number: PRJNA438545 [https://www.ncbi.nlm.nih.gov/bioproject/PRJNA438545/]. Microbial tracking-1 (MT-1) datasets (raw sequencing data and sequence assembly files) were obtained from GLDS-67, GLDS-302, GLDS-303, GLDS-309, GLDS-311 and GLDS-350. The rest of the samples are deposited at DDBJ/ ENA/GenBank or are unpublished.

## Supplementary Information

**Additional file 1: Figure S1**. Distribution of DeepARG classification probability and best-hit identity in MT-1 pure strains isolated from the ISS. The blue dashed line indicates 50% sequence identity.

**Additional file 2: Figure S2**. Gene annotations and pan-genome analysis of MT-1 scaffolds. (a) Scatterplots of number of coding sequences (CDS) found by Prokka, genome sizes, and Antibiotic resistance genes (ARGs) detected by DeepARG. (b) Frequency of genes in the core and accessory groups (soft core, shell, cloud) for ISS isolates of *E. bugandensis* (*10 strains*) and *B. cereus* (*10 strains*).

**Additional file 3: Table S1**. Rank of MT-1 isolates, ordered by number of ARGs predicted, shown in Fig. 4a. Species information obtained from Microbial Nucleotide BLAST.

**Additional file 4: Table S2**. Phenotypic antibiotic resistance testing results for *E. bugandensis* and *B cereus*.

**Additional file 5: File S1**. Raw results of the DeepARG analyses (zip compressed).

## Data Availability

The code use for the analysis is available at https://github.com/pmb59/AMRISS.

## Abbreviations

AMR: Antimicrobial resistance
ARG: Antibiotic resistance gene
AST: Antibacterial susceptibility test
ISS: International Space Station
MAG: Metagenome-assembled genome
MDR: Multi-drug resistance
MT-1: Microbial Tracking–1
PMA: Propidium monoazide
WGS: Whole-genome sequencing
