# Supplementary figures and images for: Machine learning algorithm to characterize antimicrobial resistance associated with the International Space Station surface microbiome
Source: Microbiome. 2022 Aug 24;10:134. doi: 10.1186/s40168-022-01332-w (PMC9400218; doi:10.1186/s40168-022-01332-w)

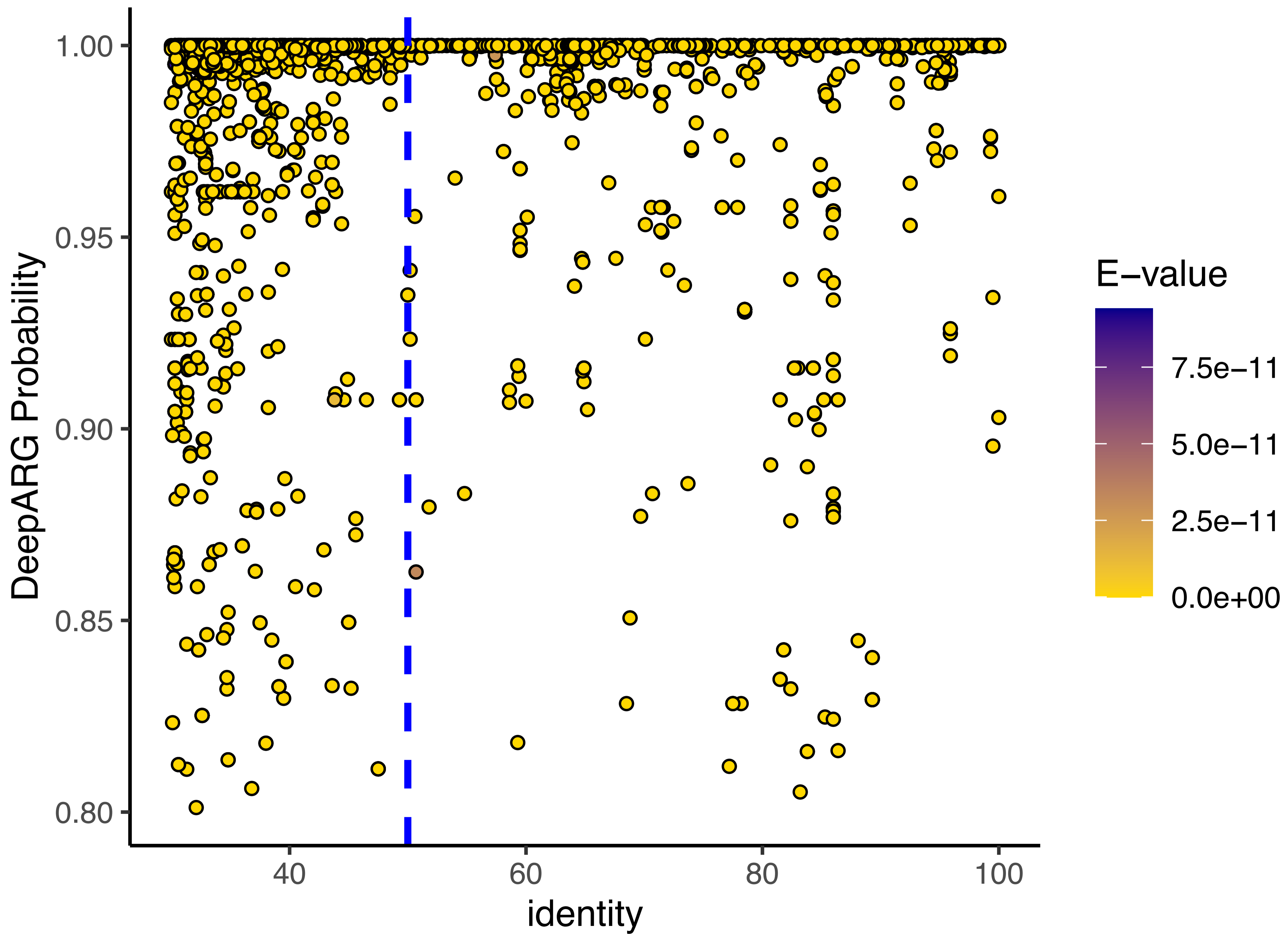

Supplement: Supplementary file 2 — Additional file 1: Figure S1. Distribution of DeepARG classification probability and best-hit identity in MT-1 pure strains isolated from the ISS. The blue dashed line indicates 50% sequence identity. [file 40168_2022_1332_MOESM1_ESM.pdf]

**a**

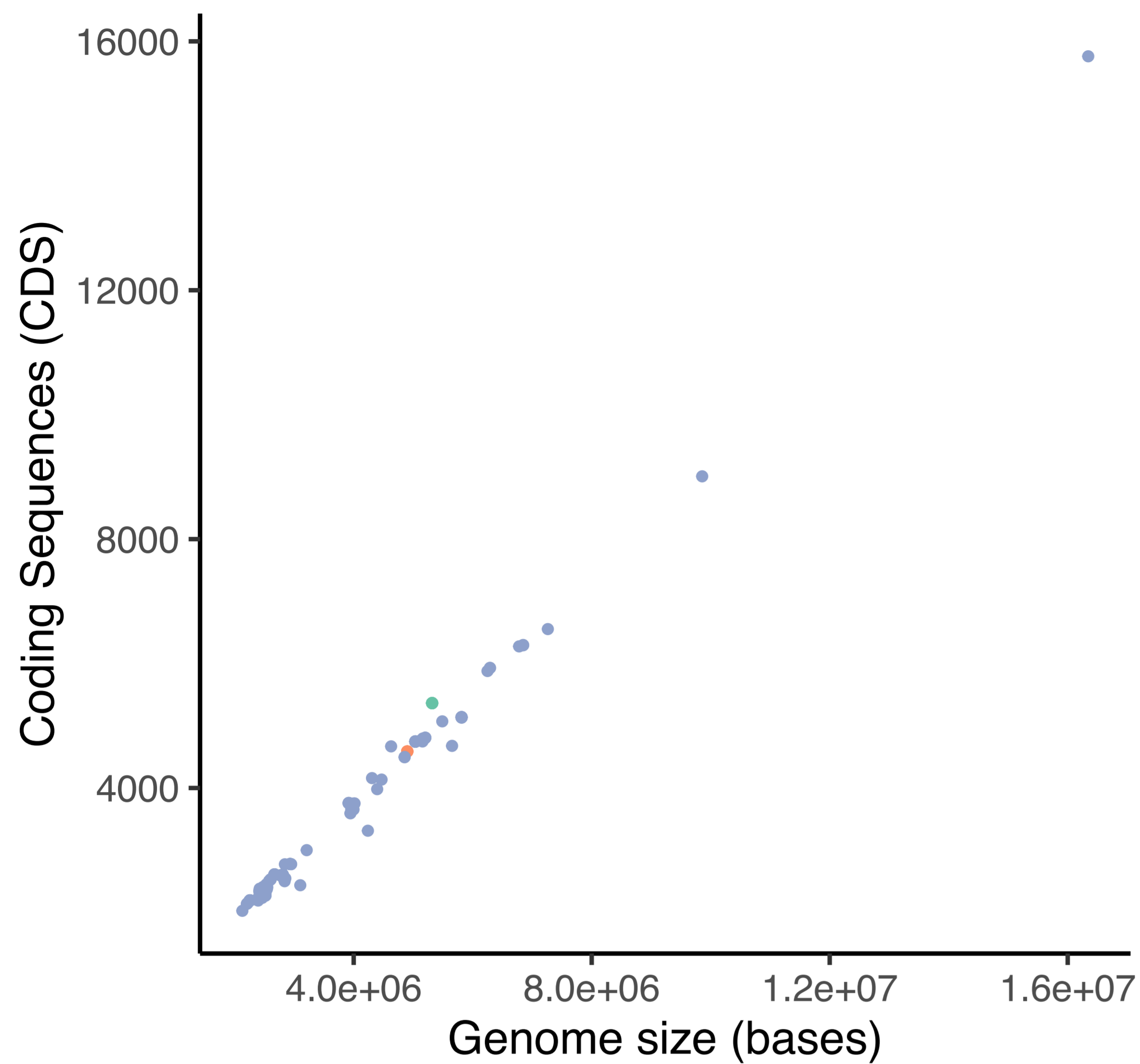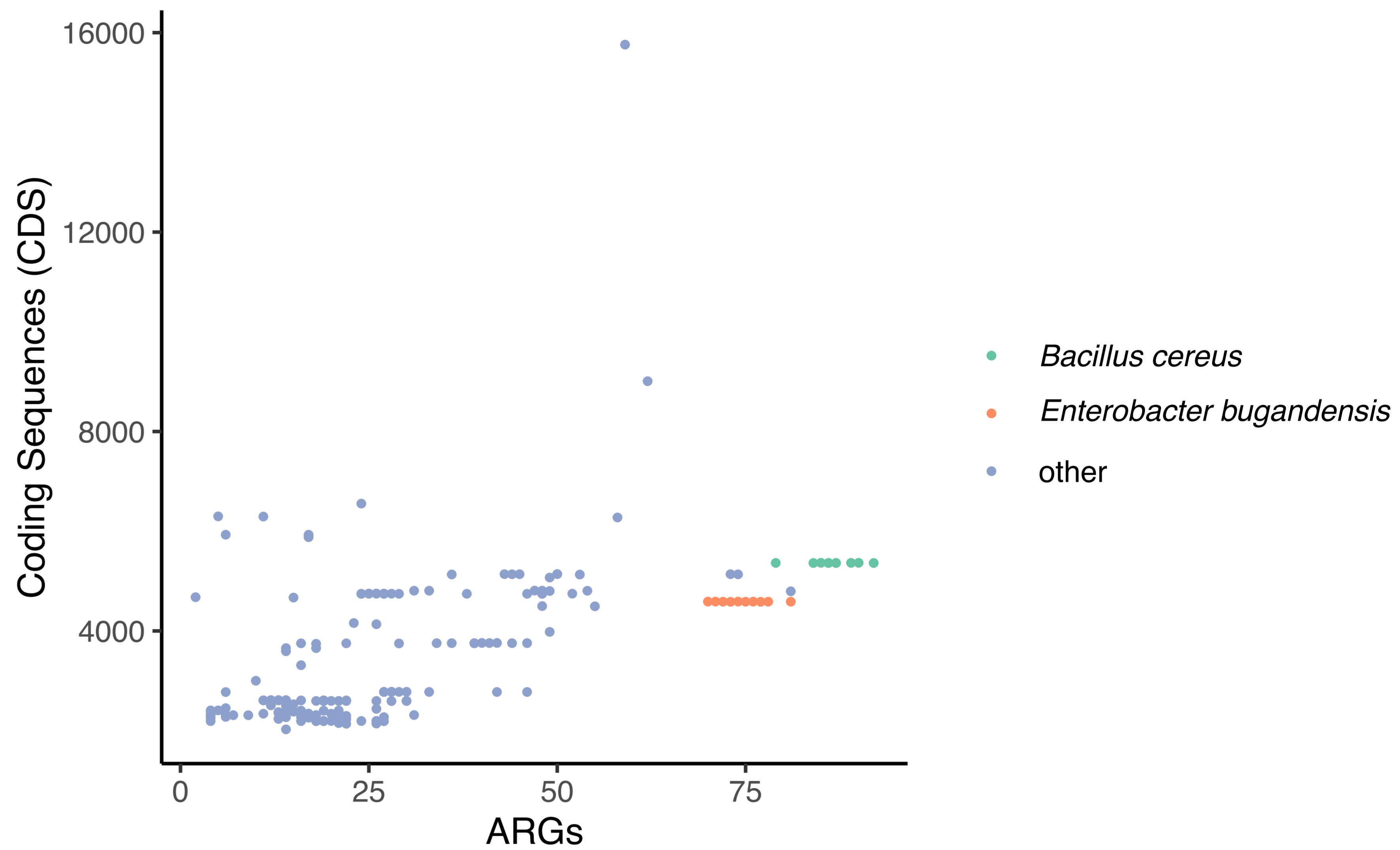

**b**

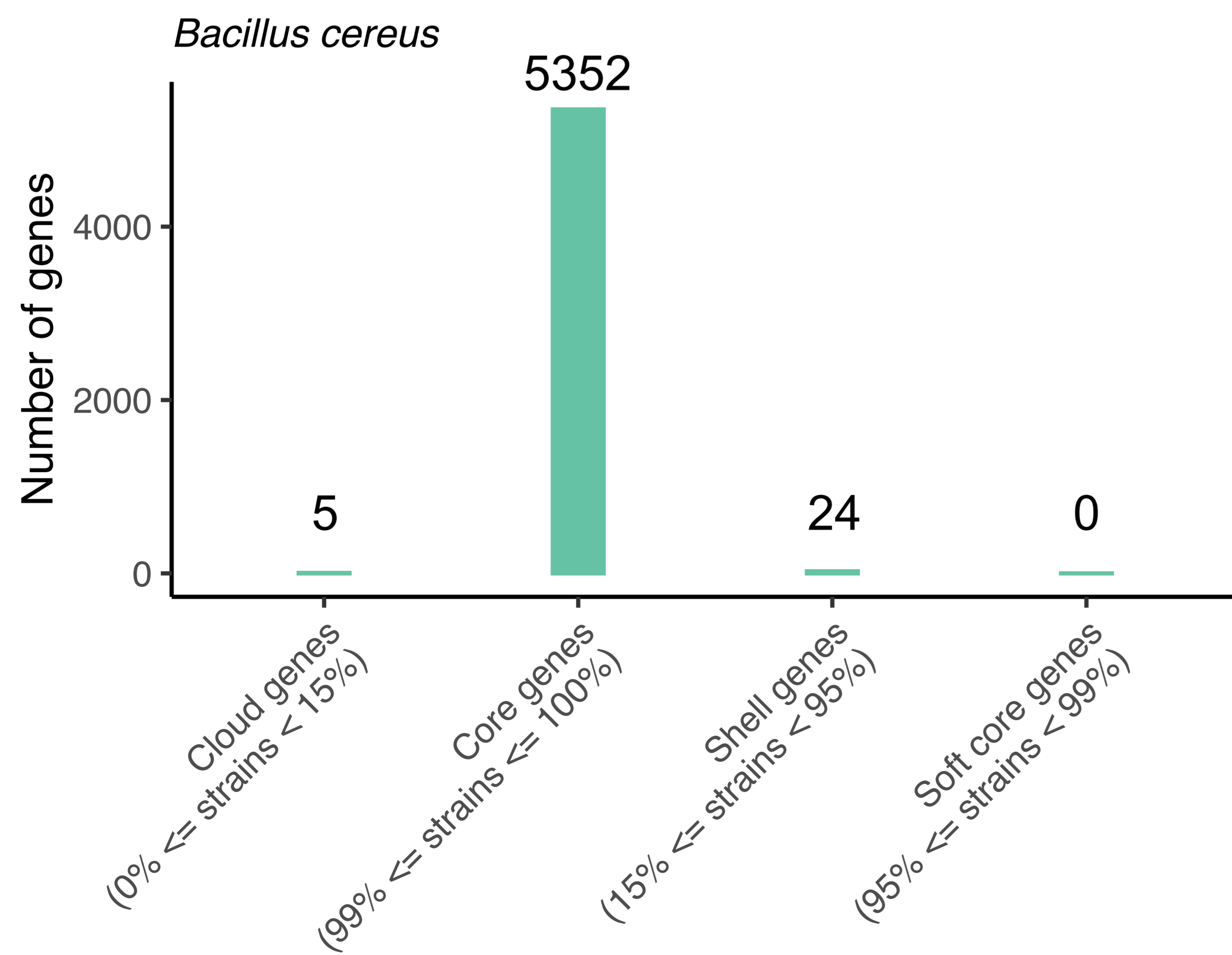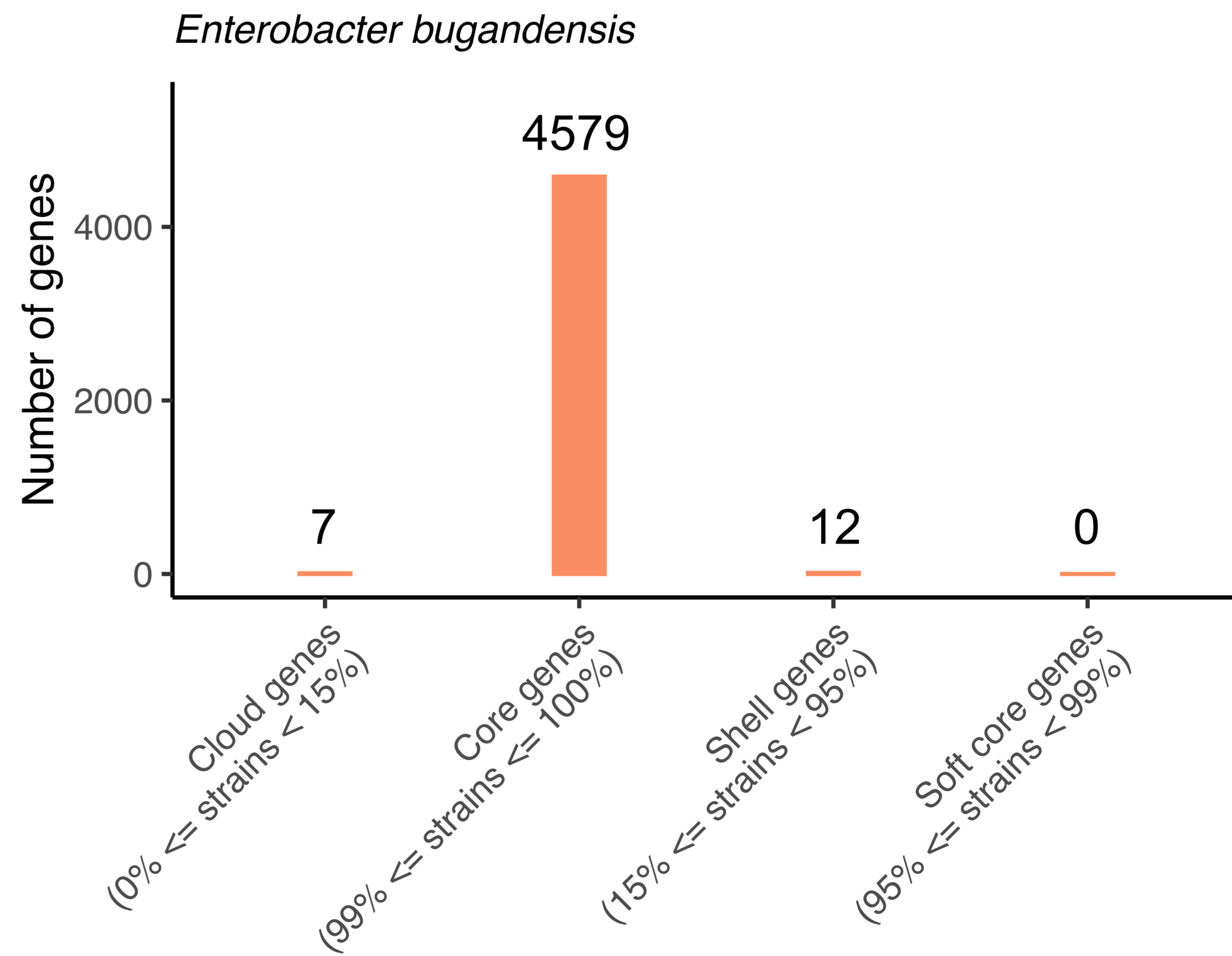

Supplement: Supplementary file 3 — Additional file 2: Figure S2. Gene annotations and pan-genome analysis of MT-1 scaffolds. (a) Scatterplots of number of coding sequences (CDS) found by Prokka, genome sizes, and Antibiotic resistance genes (ARGs) detected by DeepARG. (b) Frequency of genes in the core and accessory groups (soft core, shell, cloud) for ISS isolates of E. bugandensis (10 strains) and B. cereus (10 strains). [file 40168_2022_1332_MOESM2_ESM.pdf]
